# Chromatographic and Spectroscopic Analyses of Cannabinoids: A Narrative Review Focused on Cannabis Herbs and Oily Products

**DOI:** 10.3390/molecules30030490

**Published:** 2025-01-22

**Authors:** Céline Duchateau, Caroline Stévigny, Jehan Waeytens, Eric Deconinck

**Affiliations:** 1 Sciensano, Scientific Direction Physical and Chemical Health Risks, Medicines and Health Products Rue Juliette Wytsmanstraat, 14, 1050 Brussels, Belgium; 2 RD3-Pharmacognosy, Bioanalysis and Drug Discovery Unit, Faculty of Pharmacy, Université Libre de Bruxelles (ULB), Bld Triomphe, Campus Plaine, CP 205/5-B, 1050 Brussels, Belgium

**Keywords:** cannabinoids, cannabis herbs, oil, review, chromatography, spectroscopy

## Abstract

*Cannabis sativa* L. is cultivated nowadays for agricultural, industrial, and medicinal applications and also for recreational use. The latter is due to the presence of delta-9-tetrahydrocannabinol, a psychoactive substance. Recreational cannabis policies vary between different countries, which has led to the lack of a clearly defined legal context for cannabis and also a diversity of products derived from or containing cannabis on the (il)legal market. These cannabis-derived products have regained attention, notably because of their cannabinoid content. This review aims to assess and present analytical methods developed to analyze phytocannabinoids with spectroscopic and chromatographic techniques in specific cannabis matrices: herbs and oily products. Published papers from 2018–November 2024 were searched for with precise criteria, analyzed, and summarized. In the studies, liquid and gas chromatographic techniques (>70% reviewed papers) were the most used and have been widely applied using similar methods, and most papers were focused on cannabis herbs (>75%). Techniques were also compared and future challenges were identified. A comparison of different specificities of chromatographic and spectroscopic techniques discussed in this current review has also been established and summarized.

## 1. Introduction

*Cannabis sativa* L. is a widespread species from the Cannabaceae family that naturally occurs in various habitats from the sea to the foothills of the Himalayas. The number of species in the *Cannabis* genus has long been controversial. Some authors reported three different species: *Cannabis sativa* L., *Cannabis indica* Lam., and *Cannabis ruderalis* Janish [1]. Currently, only one species is considered to belong to the genus cannabis and includes two varieties, *sativa* and *indica.* The taxonomy is uniform and one simple and practical system of classification is based on the chemotype, considering the variety *sativa* as fibrous and the variety *indica* as narcotic [2].

The plant was first discovered in Central Asia 12,000 years ago and was mainly used for its fibers to produce ropes and nets, as well as for dietary purposes and as traditional medicine. As an example, it was used in ayurvedic medicine to treat pain, nausea, and anxiety, and also to induce euphoria. Nomadic populations spread the cannabis seeds around the world during their commercial exchanges, leading toward various discoveries and descriptions of medicinal applications, resulting in what is called ‘the golden age of medicinal cannabis’ between the 19th and 20th centuries [3].

To date, more than 177 phytocannabinoids have been identified in *Cannabis sativa* L. [4,5]. The chemical structures of some main phytocannabinoids are shown in Table 1. Phytocannabinoids are terpenophenolic compounds and are considered the main active constituents of the plant. They are biosynthesized by the glandular trichomes, particularly in stalked trichomes [6]. Decarboxylated phytocannabinoids were long assumed to be authentic natural products but, currently, it is assumed that 95% of phytocannabinoids, such as delta-9-tetrahydrocannabinol (Δ^9^-THC), cannabidiol (CBD), and cannabichromene (CBC), exist as their acid precursor form. After harvest and when exposed to heat via smoking or baking or when exposed to light, the decarboxylated phtocannabinoids are readily formed by non-enzymatic thermal decarboxylation. These factors are also responsible for the oxidation of THCA in cannabinolic acid (CBNA) and the oxidation of Δ^9^-THC in cannabinol (CBN) [7,8].

Nowadays, the plant is mainly cultivated for agricultural and industrial applications, as well as for recreational and medicinal uses.

Agricultural and industrial hemp has a wide range of applications, including as a food source for humans and animals and use in commercial products such as textiles, clothing, biodegradable plastics, paint, and so on. Hemp cultivation is also considered a green product and is recognized as such by the European Green Deal due to its light weight and durability [9]. Thanks to this Green Deal, hemp production in the European Union (EU) is flourishing, with France as the largest producer, representing 70% of the total production of the EU [9]. Farmers in Europe should have a license to cultivate hemp for industry and should use only the seventy-five varieties of *Cannabis sativa* L. listed in the common catalog of varieties of agricultural plant species [10] that can be marketed in both the EU and Switzerland.

A maximal content of delta-9-tetrahydrocannabinol (Δ^9^-THC), the psychoactive compound of cannabis, in agricultural hemp is fixed at 1% (*w*/*w*) in Switzerland [11], as opposed to the EU, where a limit of 0.3% (*w*/*w*) is applied [12]. Worldwide, every country has its own legislation and limits.

The reason for this is that cannabis today is most commonly used for recreational purposes. Indeed, the presence of Δ^9^-THC has made cannabis the most widely consumed illicit drug in Europe and one of the most popular worldwide. Here, concentrations of Δ^9^-THC are generally above 15% (*w*/*w*). Extensive developments in cannabis have been influenced by the recreational cannabis market in the United States of America and the development of “cannabis-derived products” containing extracts issued from the cannabis plant [13]. Today, cannabis, as a recreational drug, falls under legislation regarding illicit drugs, consisting of three international drug control conventions: the single convention on narcotic drugs of 1961 (amended in 1972) adopted by 154 countries [14], the convention on psychotropic substances of 1971, adopted by 184 countries [15], and the United Nations convention against illicit traffic in narcotic drugs and psychotropic substances of 1988, adopted by 191 countries [16]. In principle, these conventions do not allow countries to legalize the recreational use of cannabis, although in 2013, Uruguay was the first to legalize its production, possession, detention, and distribution [17]. In 2018, Canada followed suit and started a worldwide debate on the subject [18]. Within the European Union, several countries are developing recreational cannabis policies. For instance, in the Netherlands, its sale and use are tolerated.

Currently, it is obvious that the legal context of cannabis is not clearly defined. The scope of cannabis policies encompasses the regulation for medicinal use, for cannabis-derived products, such as cosmetics, and the control of illicit cannabis. Indeed, the diversity of products derived from or containing cannabis, as well as extracted or synthetically produced cannabinoids, is very broad. An important category includes products used for medical and medicinal purposes. Therefore, cannabis is produced by some companies for the treatment of pain, anxiety, depression, sleep disorders, and neurological disorders [19]. On the other hand, registered medicines have been launched on the market in different regions of the world. Some are based on synthetic cannabinoids [20], for example, nabilone, which is used in the treatment of anorexia and for its antiemetic effects, and dronabinol, which is used in the treatment of multiple sclerosis and pain. Others are based on *Cannabis sativa* L. extracts, used in the treatment of multiple sclerosis [21], and on naturally occurring cannabinoids, such as cannabidiol (CBD), which is applied as an adjunctive therapy for the treatment of Lennox–Gastaut or Dravet syndrome [22]. Next to these recognized medicinal products, there is a growing number of so-called “low-Δ^9^-THC products”, which are available in pharmacies, shops, via the Internet, and through illegal channels. In addition, for these products, legislation varies widely between countries, from considering them as illegal to permitting over-the-counter sales. Low-Δ^9^-THC cannabis products are numerous and Table 2 provides an overview of the different types of products available on the European market.

The wide variety of cannabis and cannabis-derived products, both existing and emerging, also necessitates market surveillance in order to protect the safety of patients and consumers. For all these products, the most important compounds of interest are cannabinoids. In this context, cannabinoids can be split into two types: phytocannabinoids, present in the plant *Cannabis sativa* L., and synthetic cannabinoids. Endocannabinoids are a third type and are molecules synthesized by the human body. Therefore, they are not within the scope of this review, since they are not used in the products discussed here.

Although Δ^9^-THC and CBD are the most-targeted cannabinoids during the analysis of these products, it is also important to monitor some other cannabinoids, e.g., cannabichromene (CBC), cannabinol (CBN, which is the Δ^9^-THC degradation product), cannabidivarin (CBDV), cannabigerol (CBG), and tetrahydrocannabidivarin (THCV), since it is known that they also often occur in products. Some products even claim to have a higher dosage of these compounds, linking them to several health claims and benefits. Phytocannabinoids, contrary to endocannabinoids, which are naturally occurring substances produced in the human body, are capable of binding to cannabinoid receptors with high affinity and have numerous other targets besides these receptors [8]. Therefore, both types of cannabinoids have the same sites of action, explaining their different effects and activities.

This review intends to provide a structured review of the chromatographic and spectroscopic techniques and methods described for the analysis of phytocannabinoids in (para)pharmaceutical cannabis-derived products, evaluating their advantages and disadvantages and emphasizing the necessity of effective method validation. Considering the wide scope of cannabis-derived products on the market, this review will focus on the two most popular matrices, i.e., herbal products and so-called CBD oils. The different techniques will be compared and future challenges will be identified. The analysis of synthetic cannabinoids is considered out of the scope of this review since in the legal market, they are present in registered medicines, with validated and approved methods in the marketing authorization files of the companies. In the illegal market, analysis falls under forensic analysis and the fight against new psychoactive substances in the illicit drug circuit. In addition, registered medicines based on naturally occurring cannabinoids were considered out-of-scope, since their analytical methods for quality control are part of confidential marketing authorization data and are product- and company-specific.

## 2. Review of the Analytical Techniques and Methods

- This section is inspired by the published thesis of Duchateau C. [23]

When using the keyword “cannabi*” (for cannabis and cannabinoids) in the Scopus database, more than 105,717 documents were found, and the distribution of these documents across the various domains is as follows: more than 63% in medicine, 23% in pharmacology, toxicology, and pharmaceutics, 6% in chemistry, and 5% in agricultural and biological sciences. Recently developed analytical techniques and methods for testing cannabinoids in herbal materials and oils were reviewed.

The increased interest in cannabis has led to a growing need for the development of qualitative and quantitative methods for the analysis of cannabinoids in many areas. Cannabis analysis is performed to control the quality of the material used, as well as to determine the difference between fiber and recreational cannabis [24]. An extensive investigation of the analytical techniques to determine cannabinoids was performed here with an emphasis on the analysis of plant materials and oils. Due to the large number of scientific publications on this topic, the literature review has been deliberately restricted to the period from 2018–2024 (November). By searching for the combination of the word “cannabi*” combined with the analytical technique of interest within titles, keywords, and abstracts and limiting the search to the “chemistry”, “pharmacology, toxicology, and pharmaceutics”, and “agricultural and biological sciences” areas, papers were found using the Scopus and Web of Science databases.

Numerous techniques have been employed for the identification and quantification of cannabinoids. Cannabinoids in plants and oils are frequently analyzed using gas chromatography (GC) and liquid chromatography (LC) [25]. Because of the current laws on Δ^9^-THC, the plant material is generally the targeted matrix. The Cannabis Analytical Science Program of the AOAC (Association of Official Analytical Collaboration) recommends other cannabinoids of interest [26].

Spectroscopic and electroanalytical methods have also been investigated. Applications based on infrared and Raman spectroscopy have shown themselves to be suitable in testing for both quantitative and qualitative purposes [27]. Indeed, these techniques use hand-held devices, which makes them interesting tools for on-site, quick, and reagent-less quality control [28]. Although mid-infrared spectroscopy (MIRS) and Raman spectroscopy have been recently applied using modern instrumentation, near-infrared spectroscopy (NIRS) is generally used for cannabis analysis [29].

## 3. Analytical Techniques

- This section is inspired by the published thesis of Duchateau C. [23]

### 3.1. Gas Chromatography (GC)

GC is a well-known and established separation technique that, when combined with a suitable detection system, enables the analysis of a wide range of analytes in complex samples. The most widely used detectors in GC, particularly in the analysis of cannabinoids, are mass spectrometry (MS) and flame ionization detection (FID). GC can be applied in the analysis of mixtures containing volatile components with a vapor pressure of a few mmHg, compounds with boiling points ranging from 0 to 425 °C, and compounds that can be heated without decomposition, such as cannabinoids [30]. Table 3 gives an overview of cannabis-related GC applications.

The flow in GC is generally between 0.5 and 1.6 mL/min and the separation is usually performed using hydrogen [31,32,33] or helium [34,35,36,37,38,39,40,41,42,43,44,45,46,47,48,49,50,51,52,53,54,55] as the carrier gas. A high proportion (95–100%) of dimethylpolysiloxane is used as the inner wall coating in fused support coated open tubular (FSCOT) capillary columns, which are the preferred type. This kind of column is commonly defined as an “ultra-inert capillary column”.

The direct determination of the acidic forms of phytocannabinoids is not possible in GC analysis. The acidic cannabinoids (thermolabile) are turned into their decarboxylated forms at the injection port, where high temperatures (~280 °C) are present. After 15 min at 150 °C, THCA is almost completely converted into Δ^9^-THC. The production of Δ^9^-THC may be maximal at 225 °C, while decarboxylation of CBDA is already complete at about 110 °C. Only the quantification of the total form (acidic and basic form, e.g., total Δ^9^-THC) is possible with the implementation of GC, which is an advantage, e.g., in the context of Δ^9^-THC content determination in agricultural hemp [56,57,58,59]. Indeed, EU legislation only limits the total Δ^9^-THC content; therefore, it recommends methods based on GC [60]. When the determination of the acidic forms is not necessary, a heating step can be implemented. However, it should be kept in mind that a significant loss of components could be caused by the high temperatures of the injector and detector. In addition to the high temperatures, the geometry of the injector port also influences the decarboxylation rate. If an accurate estimation of both decarboxylated and acidic cannabinoid forms is required using GC, a derivatization step is recommended [56,57,58,59]. Cardenia et al. have compared different silylation reactions of cannabinoids to methylation with diazomethane. This solvent was demonstrated to be better than silylation solvents but their commercial unavailability and unsuitability for routine procedures have led to silylation being the best derivatizing method [53]. Derivatization by silylation also improves peak symmetry and method sensitivity [42].

Choosing the internal standard appears to be crucial. 5α-cholestane [41,53], 4-androstene-3,17-dione [42], squalane [50], or a deuterated standard [33,47,48,49] are examples of potential internal standards. The comparison between two internal standards was achieved in the development of the GC-MS method by Cardenia et al., and it appears that sensitivity is improved with 5α-cholestane compared to the deuterated (D) standard [53]. The recovery values range from ±15% to 20%. The limit of detection (LOD) and quantification (LOQ) are generally in the microgram range, although nanogram or picogram ranges could be attained.

The accuracy of quantitative results is correlated with the extraction step, which is a crucial step in cannabinoid analysis. Solvent-based methods are generally used to extract cannabinoids from herbal samples. Methods using apolar solvents (e.g., n-hexane [41,43,49,51], dichloromethane (DCM) [55], acetone [39], or diethyl ether [47,48]), polar solvents (e.g., methanol (MeOH) [31,33,36,38,46] or ethanol (EtOH) [35,37,45,46] alone), or the combination of different solvents [42,53] were developed. The extraction processes are more complex for oily samples. For instance, QuEChERS is used for sample clean-up in order to avoid the introduction of an oily matrix into the GC port [43].

Quite recently, similar methods were developed in order to simultaneously analyze cannabinoids and terpenes, which are both important in the quality control of cannabis and cannabis-derived products [36].

**Table 3:** GC applications: overview of the literature.

Analytical Technique 1st Author [Reference]	Matrix (Sample State)	Cannabinoids	Internal Standard	Column	Carrier Gas Flow Rate/Velocity	GC/Detector Conditions Temperature in °C	Extraction Solvent (Recovery Rates After Extraction)	Derivatization	Analysis Time Quantitative (LOQ)/Screening (LOD)
2024									
Two-dimensional GC-MS Spadafora N. [34]	Dried inflorescences	CBDV, CBD(A), CBC, CBG(A), Δ^9^-THC(A)	No	(1°) HP-5-ms (0.18 µm, 20 m × 0.18 mm) (2°) DB-17MS (0.25 µm, 2.5 m × 0.25 mm)	Helium (1°) 0.5 mL/min (2°) 10 mL/min	T_o_: 40–230 T_i_: 250 T_s_: -	SPME	no	n.m. Quantification (n.m.)
GC-FID Micalizzi G. [36]	Dried, pulverized, and sieved inflorescences	CBD(A), Δ^9^-THC(A)	n-nonadecane	HP-5 (0.25 µm, 15 m × 0.25 mm)	Helium 1.0 mL/min	T_o_: 240 T_i_: 290 T_FID_: 300	MeOH	no	8 min Quantitative (n.m.)
GC-FID Arsenault T. [31]	Dried, sieved, and mixed flowers (buds)	CBD, Δ^9^-THC	No	Rxi-35sil msS (0.25 µm, 15m × 0.25 m)	Hydrogen 4 mL/min	T_o_: 225–325 T_i_: 250 T_FID_: 350	MeOH	no	10 min Quantitative (n.m)
2023									
GC-MS Koo Y. [37]	Dried and ground plant material (flower, stem, root, and leaves)	CBD, Δ^9^-THC	no	DB-5-ms (0.25 µm, 15 m × 0.25 mm)	Helium 1.0 mL/min	T_o_: 80–300 T_i_: 300 T_s_: -	EtOH	no	<24 min. Quantitative (n.m.)
GC-MS Motiejauskaite D. [38]	Dired and ground inflorescences	CBDVA, CBL, CBD, CBC, CBN, CBG	no	Rxi-5 ms (0.25 µm, 30 m × 0.25 µm)	Helium	T_o_: 110–280 T_i_: 250 T_s_: 200 Electron ionization	MeOH, Triton-X-100 (>86%)	no	39 min Quantitative (n.m.)
GC-MS Ronald H. [39]	Dried ground inflorescences	CBD, THC, CBN	no	Elite-5ms	Helium (0.8 mL/min)	T_o_: 200–280 T_i_: 280 T_s_: 225 Electron ionization	Acetone	no	45 min Quantitative (n.m)
GC-MS Judžentienė A. [40]	Inflorescence, leave, root, and stem	CBC, CBD(A), CBG, CBN	no	Rxi-5ms (0.25 µm, 33 m × 0.25 mm)	Helium 1 mL/min	T_o_: 50–250 T_i_: 250 T_s_: 220 EI ionization	MeOH	no	47 min Qualitative
GC-FID Gul W. [42]	Dried and ground inflorescences	CBC(A), CBL(A), CBD(A), CBDV(A), CBG(A), CBN(A), THCV, Δ^8^-THC, Δ^9^-THC(A), Δ^9^-THCV(A))	4-androstene-3,17-dione	DB-1MS (0.25 µm, 15 m × 0.25 mm)	Helium 0.8 mL/min	T_o_: 190–300 T_i_: 275 T_FID_: 300	ACN:MeOH	BSTFA	17.5 min Quantitative (LOD: 0.1 µg/mL LOQ: 0.25–0.50 µg/mL)
2022									
GC-FID Wilson J. [32]	Dried sieved inflorescences	CBD	no	Rxi-35Sil MS (0.25 µm × 15 m × 0.25 mm)	Hydrogen 1.75 mL/min	-	EtOH (>63%)	no	n.m. Quantitative (n.m.)
GC-MS De Prato L. [41]	Dried ground inflorescences	CBC, CBD(A), CBDV, CBG(A), Δ^8^-THC(A), Δ^9^-THC(A)	5α-cholestane	HP-5MS (0.25 µm, 15 m × 0.25 mm)	Helium 1.2 mL/min	T_o_: 80–300 T_i_: - T_s_: 280 EI ionization	n-Hexane	MSTFA BSTFA	n.m. Semi-quantitative (LOD: 82.31–166.40 mg/kg LOQ: 274.36–554.65 mg/kg)
2021									
GC-MS Ahmed A.Q. [33]	Dried ground flowers	CBC CBD CBG CBL CBN Δ^9^-THC	CBD-d3, Δ^9^-THC-d3	HP-5MS capillary column (0.25 µm, 30 m × 0.25 mm)	Hydrogen 1.6 mL/min	T_o_: 180–250 T_i_: 280 EI ionization	MeOH (80–100%)	no	14 min Quantitative (LOD: 0.006–0.008 mg/mL LOQ: 0.018–0.026 mg/mL) (SIM mode)
GC-MS Duchateau C. [43]	Oils	CBN, CBDV, CBT, CBC, Δ^8^-THC, Δ^9^-THC, THCV, CBG	methylarachidate	VF-5 MS (0.25 µm, 30 m × 0.25 mm)	Helium 1.5 mL/min	T_o_: 200–280 T_i_: 250 T_s_: 280 EI ionization	n-hexane QuEChERS (Bond Elut EMR lipid)	no	17.3 min Screening (LOD: 10–14 ng/mL) Quantitative (n.m.)
2020									
GC-FID Zekič J. [50]	Dried and ground plant material	CBC, CBD, CBG, CBN, Δ^8^-THC, Δ^9^-THC	squalane	RTX-50 (0.25 µm, 30 m × 0.25 mm)	Helium 2 mL/min	T_o_: 60–290 T_i_: 310 T_FID_: 310	Acetone (>92%)	no	17 min Quantitative (LOD: 0.662–0.857 µg/mL LOQ: 2.207–2.858 µg/mL)
GC-MS Slosse A. [35]	Dried ground inflorescences	THCV, CBD, CBC, Δ^9^-THC, CBN, CBG	tribenzylamine	DB5-ms (0.25 µm, 15 m × 0.25 mm)	Helium 1.3 mL/min	T_o_: 60–320 T_i_: 230 T_s_: - EI ionization	EtOH	no	29 min Qualitative (n.m.)
Two-dimensional GC-TOF-MS (low resolution)	Dried inflorescences	CBD, CBN, Δ^9^-THC	chlorobenzene-d5	Two MXT Y unions Nonpolar Rxi-5MS (0.25 µm × 25 m × 25 mm) Midpolar Rxi-17Sil MS (0.25 µm × 5 m × 0.25 mm)	Helium 0.4 mL/min 7 mL/min	T_o_: 50–330 T_i_: 20–300 T_s_: 230	MeOH Acetone Water	no	n.m. Quantitative (LOD: 0.02–0.15 µg/mL LOQ: 0.05–0.51 µg/mL)
Two-dimensional GC-TOF-MS (high resolution) Franchina F. [44]					Helium 1 mL/min	T_o_: 50–330 T_i_: 20–300 T_s_: 250		no	
GC-FID Bakro F. [45]	No dried ground leaves and inflorescences	CBD	n-tridecane	RTX-5 0.1 µm × 10 m × 0.1 mm)	Helium 46 cm/s	T_o_: 60–310 T_i_: 310 T_FID_: 340	EtOH	no	16 min Quantitative (LOD: 0.16 µg/mL LOQ: 0.55 µg/mL)
GC-FID Baranauskaite J. [46]	Dried and ground inflorescences	CBD, CBG	/	Rxi-5MS (0.25 µm × 30 m × 0.25 mm)	Helium 1 mL/min	T_o_: 80–310 T_i_: 290 T_FID_: 330	EtOH	no	30 min Quantitative (LOD: 0.21–0.25 µg/mL LOQ: 0.66–0.75 µg/mL)
GC-MS Fernandez N. [47,48]	Oils	CBC, CBDA, CBD, CBG, CBN, THCA, Δ^9^-THC	Δ^9^-THC-d3	HP-5MS (0.25 µm, 30 m × 0.25 mm)	Helium 1 mL/min	T_o_: 60–300 T_i_: 280 T_s:_ 280 EI ionization	Diethyl ether	MSTFA	26 min Screening Quantitative (n.m.) LOQ: 0.04–0.1 µg/mL)
GC-FID Duchateau C. [55]	Dry flowers crushed by hand	CBN, Δ^9^-THC	methylarachidate	DB-5ms (0.25 µm × 30 m × 0.25 mm)	Helium 1.5 mL/min	T_o_: 270–310 T_i_: 225 T_FID_: 300	DCM	no	(n.m.) Quantitative (n.m.)
GC-MS ElSohly M. [49]	Oils	CBD(A), Δ^9^-THC(A)	CBD-d3, Δ^9^-THC-d3	D-1 (0.4 µm, 10 m × 0.18 mm)	Helium 0.4 mL/min	T_o_: 180–280 T_i_: 250 T_s:_ -	n-Hexane	MSTFA	13 min Quantitative (LOD: 1 µg/mL LOQ: 2.5 µg/mL)
GC-TOF/MS Delgado-Povedano M.M. [51]	Dried and ground leaves and inflorescences	CBC, CBD, CBDVA, CBDV, CBG, CBL, CBN, THCA, Δ^8^-THC Δ^9^-THC, THCV	no	DB-5MS-UI (0.25 µm, 30 m × 0.25 mm)	Helium 1 mL/min	T_o_: 50–310 T_i_: 250 T_s:_ 305 EI ionization	n-Hexane	BSTFA TMCS Pyridine	37 min Screening (n.m.)
2019									
GC-MS Burnier C. [52]	Cannabis plant (flowers and leaves)	CBD, CBN, Δ^9^-THC	tribenzylamine	HP-5MS (0.25 µm, 30 m × 0.25 mm)	Helium 1 mL/min	T_o_: 50–260 T_i_: 280 T_s:_ 230	MeOH EtOH	no	15 min Quantitative (LOD: 4.54 µg/mL LOQ: 15.13 µg/mL)
2018									
GC-MS Cardenia V. [53]	Dried flowers and leaves	CBC, CBD, CBDA, CBG, CBGA, CBN, THCV, Δ^8^-THC, Δ^9^-THC, THCA	5α-cholestane	Restek RTX 5 (0.1 µm,10m × 0.1 mm)	Helium n.m.	T_o_: 180–250 T_i_: 300 T_s_: 200 EI ionization	MeOH :CHCl_3_	Methylation: diazomethane Silylation: pyridine, MSTFA-TMCS, n-hexane	10 min Quantitative (LOD: 2.16–58.86 ng/mL LOQ: 7.18–169.29 ng/mL)
GC-MS Fodor B. [54]	Dried and ground inflorescences	CBC, CBD, CBG, CBN, Δ^9^-THC, 11-OH-THC, THCA-A	no	HP-5MS capillary column (0.25 µm × 30 m × 0.25 mm)	Helium 1 mL/min	T_o_: 100–300 T_i_: 300 T_s_: 210	MeOH	BSTFA TMCS Pyridine MTBSTFA TBDMCS TMCS	20 min Quantitative (LOQ: 20–80 pg/µL)

ACN: acetonitrile; BSTFA: N,O-Bis (trimethylsilyl)-trifluoroacetamide; CBC(A): cannabichromen(-ic acid); CBD(A): cannabidiol(-ic acid); CBDV(A): cannabidivarin(-ic acid); CBG(A): cannabigerol(-ic acid); CBL(A): cannabicyclol(-ic acid); CBN(A): cannabinol(-ic acid); CBT: cannabicitran; HMDS: hexamthyldisilazane; LOD: limit of detection; LOQ: limit of quantification; Δ^9^-THC(A): Δ^9^-tetrahydrocannabinol(-ic acid); Δ^8^-THC: Δ^8^-tetrahydrocannabinol; THCV(A): tetrahydrocannabidivarin(-ic acid); MTBSTFA: N-methyl-N-ter.-butyl dimethylsilyltrifluoroactamide; MSTFA: n-methyl-n-trimethylsilylfrifluoroacetamide; n.m.: not mentioned; SPME: solid-phase microextraction; TBDMCS: tert. butyl dimethylchlorosilane; TMCS: trimethylchlorosilane; T_o_: oven temperature; T_i_: injector temperature; T_s_: source temperature for MS detection; T_FID_: detector temperature for FID.

### 3.2. Liquid Chromatography (LC)

For the analysis of cannabinoids, high-performance (or high-pressure) liquid chromatography (LC) performs similarly to GC. They both present a number of environmentally unfriendly issues. On the one hand, GC uses expensive gases such as helium, and on the other hand, conventional HPLC needs large amounts of organic solvents and generates a lot of waste [61]. However, HPLC and ultra-HPLC (UHPLC) do not require heating or derivative steps and are useful alternatives for analyzing the acidic form of cannabinoids [62].

In LC, the solution is directly injected at room temperature into the mobile phase at the head of the chromatographic column. Sample components are separated through the differences in interaction between the stationary phase and the mobile phase (flowing liquid), and eluted molecules are detected at different retention times (Rt) at the outlet of the column [63,64].

LC is used in conjunction with different detectors. Mass spectrometers (MS), ultraviolet–visible detectors (UV–vis), and diode array detectors (DAD) are the most widely used in the context of analyzing natural cannabinoids. Methods described for the determination of cannabinoids in various cannabis matrices such as plants, extracts, cannabis oils, hemp food products, and so on are based on (UHP)LC-MS/MS and (UHP)LC-DAD (UV) [62].

Table 4 gives an overview of cannabis-related LC applications.

As for GC analysis, the extraction is a crucial step in obtaining accurate cannabinoid quantification. It can be observed that cannabinoids in plant materials are generally extracted by solvent-based methods using acetonitrile (ACN) [65,66,67,68,69,70], MeOH [34,71,72,73,74,75,76,77,78,79,80,81,82,83], EtOH [52,84,85,86,87,88] pentane [40], isopropanol [59,89,90], and acetone [91], or by a combination of different solvents [92,93,94,95,96,97,98]. The recovery of the extraction step [73] is sometimes mentioned in different papers and is generally higher than 70%. In an ecological context, methods using low solvent quantities, such as ultrasound-assisted solid–liquid extraction, were developed [97].

Internal standards are generally used, such as deuterated compounds [71,89,90,91,99], but also abnormal CBD [74], cannabichromeorcin [74], and other molecules, e.g., phemprocoumon [70], fencamfamine [94], tridecane [93], ibuprofen [84], and tribenzylamine [52]. Some authors did not use an internal standard for the quantification of cannabinoids.

The physico-chemical properties of cannabinoids are similar and it is a challenge to separate them under isocratic conditions. UHPLC is used in most applications to reduce the time needed for analysis. Reversed-phase C18-packed columns (or less commonly, C8-packed columns) with gradient or isocratic elution have shown the best performance for cannabinoid determination. Mobile phases composed of different proportions of ACN, MeOH, water, either pure or with a small percentage of formic acid or acetic acid, and various acetate, ammonium, and formate buffers are typically used for elution. Quite recently, similar methods were developed in order to simultaneously analyze cannabinoids and terpenes, which are both important in the quality control of cannabis and cannabis-derived products [65,88]. A relatively recent method allowing the simultaneous analysis of terpenes and cannabinoids was developed using two-dimensional liquid chromatography coupled with smart active modulation, which allows the simultaneous determination of different concentration levels in complex samples [65]. In the context of sustainability, the use of ultrasound-assisted extraction using eutectic solvents [100] and nano-liquid chromatographic systems was explored [87].

UV is commonly used since cannabinoids contain chromophores in their structure [84,94,96,101]. It can be used as a single detector (quantification in the order of µg/mL) or combined in series with MS/MS (quantification in the order of ng/mL) [97,102]. It is possible to use a quadrupole MS detector alone [67] or in combination with TOF [92]. In addition, applications using QTRAP detectors showed sensitivities in the range of pg/mL [83,93]. Compared to MS/MS detectors, UV detectors lack specificity and are approximately 100 times less sensitive than MS/MS, which provides enough sensitivity and specificity to quantify all quasi-cannabinoids. As a result, the MS/MS detector is commonly used, and electrospray (ESI) and atmospheric pressure chemical ionization (APCI) are generally encountered as ionization methods.

**Table 4:** LC applications: overview of the literature.

Analytical Technique 1st Author [Reference]	Matrix (Sample State)	Cannabinoids	Internal Standard	Analysis Time Quantitative (LOQ)/Screening (LOD)	Solvent Extraction (Recovery After Extraction)	Mobile Phase	Column (Particle Size, Length × Inner Diameter) Temperature (T°) in °C
2024							
UHPLC-UV (DAD) (228 and 306 nm) Spadafora N. [34]	Dried inflorescences	CBDV, CBD, CBDA, CBC, CBG, CBGA, Δ^9^-THC, Δ^9^-THCA	no	n.m. Quantification (n.m.)	MeOH	Water + Orthophosphoric acid (pH 2.2) ACN (gradient)	Raptor ARC-18 column (2.7 µm, 150 mm × 2.1 mm) T°: 25
2D-HPLC-DAD (200 nm) Caruso S.J. [65]	Dried inflorescences	CBGA, CBG, CBDA, CBD, CBC, THCV, CBN, Δ^8^-THC, Δ^9^-THC, THCA-A	no	75 min Screening (n.m.)	can	D1: Water + formic acid 0.05% MeOH + formic acid 0.05% (gradient) D2: Water + formic acid 0.05% ACN + formic acid 0.05% (gradient)	Zorbax SB-CN (5 µm, 250 mm × 4.6 mm) and Poroshell 120-SB C18 (2.7 µm, 50 mm × 2.1 mm) T° D1: 35 T° D2: 75
UHPLC-DAD (270 nm) Mastellone G. [100]	Died ground inflorescences and oils	CBD, CBDA	no	56 min Quantification (LOD: 0.03–1 µg/mL LOQ: 0.1–4 µg/mL)	Eutectic solvents: [Ch+] [Br-] + thymol	Water + formic acid 0.1% ACN + formic acid 0.1% (gradient)	Ascentis Express C18 (2.7 µm, 150 mm × 2.1 mm) T°: 30
UHPLC-Q-ToF-MS Woźniczka K. [92]	Fresh plant material	Δ^9^-THCA, CBDA, CBGA, CBVA, THCVA	Phemprocoumon	6 min Quantification (n.m.)	MeOH/isopriopanol 50/50 *v*/*v*	ACN + 0.1% formic acid (gradient)	Poroshell 120 PFP (2.7 µm, 100 mm × 2.1 mm) T°: 33
UHPLC-QTrap-MS Wishart D.S. [93]	Dried ground inflorescences	CBDV, CBDVA, THC-COOH, CBLA, CBCA, CBNA, CBDA, THCV, CBGA, CBD, CBN, CBC, CBG, CBL, Δ^9^-THC, THCA	Tridecane	9.5 min Quantification (LOD: 0.001–0.00426 ng/mL LOQ: 0.00333–0.0142 ng/mL)	Hexane/MeOH 3/1 *v*/*v*	0.2% formic acid 0.2% formic acid + ACN (gradient)	Zorbax Eclipse XDB C18 column (3.5 µm, 100 mm × 3.0 mm) T°: 50
HPLC-APCI-MS/MS Raeber J. [88]	Dried ground flowers	CBDV, CBG, CBD, CBDA, CBN, Δ^9^-THC, THCA (+ terpenes)	no	28 min Quantification (n.m.)	EtOH	2 mM ammonium acetate + 0.1% formic acid 2 mM ammonium acetate + 0.1% formic acid/methanol (5/95) (gradient)	Symmetry C18 (3.5 µm, 100 mm × 4.6 mm) + guard column T°: 45
UHPLC-MS/MS Cai Y. [71]	Ground flowers and leaves	CBC, CBDV, CBD(A), CBG(A), CBL, CBN, THCV, Δ^8^-THC, Δ^9^-THC, THCA-A	CBD-d3	11 min Quantification	MeOH	Water + 0.1% formic acid ACN (gradient)	Acquity BEH-C18 (1.7 µm, 2.1 mm × 50 mm) T°: 30
UHPLC-MS/MS Lindekamp N. [91]	Oils	CBC(A), CBD(A), CBDV(A), CBG(A), CBL(A), CBN(A), Δ^9^-THC(A)	CBD-d3, CBN-d3, Δ^9^-THC-d3, THCA-d3	18 min Quantification (LOD: 0.02–4.32 ng/mL LOQ: 0.07–14.38 ng/mL)	Acetone	Water + 0.1% form acid ACN + 0.1% formic acid (gradient)	Acquity UPLC BEH C18 (1.7 µm, 150 mm × 2.1 mm) T°: 30
LC-DAD Song L. [74]	Ground flowers	CBC(A), CBD(A), CBDV(A), CBG(A), CBN(A), Δ^8^-THC, Δ^9^-THC, THCA, THCV(A), CBL(A), CBT	Abnormal-CBD, cannabichromerorcin	32 min Quantification (LOQ: 0.04 µg/mL)	MeOH	Water + 0.1% formic acid + ammonium formate 0.5 mM (pH3) MeOH + ACN (isocratic)	Restek Raptor ARC-18 (2.7 µm, 150 mm × 2.1 mm) + guard column T°: 30
LC-DAD Wilson W.B. [75]	Dried ground plant (and other matrices, e.g., hemp seed oil)	CBC(A), CBDV(A), CBD(A), CBG(A), CBL(A), CBN(A), THCV(A), Δ^9^-THC, Δ^8^-THC, 9S-Δ^10^-THC, 9R-Δ^10^-THC, exo-THC	no	8 min Quantification	MeOH	ACN Water (gradient)	NexLeaf CBX for Potency C18 column (2.7 µm, 150 mm × 4.6 mm) T°: 40
2023							
UHPLC-Qtrap-MS Kanabus J. [83]	Fresh and dried ground inflorescences	CBDV, CBDVA, THC-COOH, CBLA, CBCA, CBNA, CBDA, THCV, CBGA, CBD, CBN, CBC, CBG, CBL, Δ^8^-THC, Δ^9^-THC, Δ^9^-THCV, Δ^9^-THCVA, THCA	no	10 min Quantification (LOD: 0.00003–0.005 µg/mL LOQ: 0.0001–0.02 µg/mL)	MeOH (>90%)	0.02% formic acid in ACN/5 mM Ammonium formate (gradient)	C18-Cortecs (1.6 µm, 100 mm × 2.1 mm) T°: 20
HPLC-DAD-ToF-MS Judžentienė A. [40]	Dried ground inflorescences, leaves, seeds, and roots	CBD, CBDA, CBN	no	34 min Quantification (n.m.)	Pentane	ACN + 0.1% formic acid (gradient)	Zorbax Eclipse XDB (5 µm, 150 mm × 4.6 mm) T°: 35
HPLC-DAD Correia B. [70]	Dried ground flowers and oils	CBD(A), CBN, Δ^8^-THC, Δ^9^-THC, THCA	Phemprocoumon	30 min Quantification (LOD: 0.125–0.250 µg/mL LOQ: 0.5 µg/mL)	ACN	ACN Water + 0.1% formic acid (pH 2.8)	Kinetex C18 (2.6 µm, 150 mm × 2.1 mm) + guard column T°: 20
HPLC-MS Duzan B. [67]	Oils	CBC, CBG(A), CBD(A), CBDV, CBN, Δ^8^-THC, Δ^9^-THC, THCA	no	13 min Quantification (LOD: 5–25 ng/mL LOQ: 10–50 ng/mL)	ACN (86–110.88%)	Water + 0.1% formic acid ACN + formic acid 0.1% (isocratic)	Acquity UPLC BEH C18 (1.7 µm, 100 mm × 2.1 mm) + guard column T°: 45
UHPLC-MS/MS Fabresse N. [95]	Flowers	CBD, CBN, Δ^9^-THC	CBD-d3, CBN-d3, Δ^9^-THC-d3	<6 min Quantification	Heptane:ethyl acetate (7:1)	Water+ formic acid 0.1% ACN + formic acid 0.1% (gradient)	Luna Omega Polar C18 (1.6 µm, 50 mm × 2.1 mm) T°: 40
2022							
HPLC-TOF/MS Hewavitharana A.K. [84]	Dried ground inflorescences	CBDA, CBD, CBDV, CBGA, CBG, CBN, THCA, Δ^9^-THC, THCVA, THCVA	Ibuprofen	40 min Quantification (LOD: 1.18–9.11 µg/g) LOQ: 3.93–25.3 µg/g)	EtOH	Water + MeOH + formic acid 0.1% ACN + formic acid 0.1% (gradient)	Poroshell C18 (2.7 µm, 150 mm × 2.1 mm) T°: 30
HPLC-MS/MS Hall D.R. [76]	Dried sieved inflorescences	CBCA, CBC, CBDA, CBD, CBDVA, CBDV, CBGA, CBG, CBL, CBNA, CBN, Δ^8^-THC, THCA, Δ^9^-THC, THCVA, THCV	no	8 min Quantification (LOD: 20–78 µg/g LOQ: 60–238 µg/g)	MeOH	Water + formic acid 0.1% ACN + formic acid 0.1% (gradient)	Luna Omega C18 (1.6 µm, 150 × 2.1 mm) T°: 40
UHPLC-MS/MS McRae G. [96]	Dried ground flowers	CBC(A), CBD(A), CBDV(A), CBG(A), CBL(A), CBN(A), Δ^8^-THC, Δ^9^-THC, THCA, THCV(A)	CBD-d3, CBN-d3 Δ^9^-THC-d3	21 min Quantification (LOQ: 10ng/mL)	MeOH:water (8:2) (>98.75%)	Water + formic acid 0.1% ACN + formic acid 0.1% (gradient)	Ace-3 C18-Amide (3 µm, 100 mm × 2.1 mm) + guard column T°: 40
UHPLC-DAD Song L. [77]	Hemp concentrate	CBC(A), CBDV(A), CBD(A), CBG(A), CBL(A), CBN, CBT, THCV(A), THCA, Δ^8^-THC, Δ^9^-THC	no	15 min Quantification (LOQ: 0.02 µg/mL)	MeOH	Water (pH 3.6) + formic acid 0.1% ACN (isocratic)	Luna Omega Polar C18 (1.6 µm, 150 mm × 2.1 mm) T°: 30
UHPLC-DAD (228 nm) Duchateau C. [78]	Dried and sieved aerial parts	CBD(A), CBN, Δ^9^-THC, THCA	no	11 min Quantification (LOD: 0.01–0.03% *w*/*w* LOQ: 0.03–0.2% *w*/*w*)	MeOH	Water + formic acid 0.1% ACN (isocratic)	CORTECS Shield RP18 (1.6 µm, 100 mm × 2.1 mm) T°: 35
DART-MS	Oils	CBD	CBD-d3	n.m Quantification (n.m.)	MeOH	/	/
LC-MS Huber S. [79]				n.m. Quantication (LOD: 0.657 mg/L LOQ: 1.63 mg/L)		Water + formic acid 0.1% ACN (gradient)	XSelect CSH C18 (2.5 µm, 150 mm × 4.6 mm) T°: 60
LC-MS/MS Johnson E. [68]	Oils	Δ^9^-THC	Δ^9^-THC-d9	14.25 min Quantification (n.m.)	ACN (>96%)	Water + formic acid 0.1% ACN (gradient)	Kinetex C8 (2.6 µm, 100 mm × 2.1 mm)
HPLC-MS/MS Plamieri S. [80]	Dried ground inflorescences	CBC(A), CBD(A), CBG(A), CBN, Δ^9^-THC, THCA, THCV	no	8 min Quantification (10 cannabinoids) Screening (26 cannabinoids) (n.m.)	MeOH	Water + 5 mM formic acid ACN + 5 mM formic acid (gradient)	Kinetex C18-XB (2.6 µm, 100 µm × 2.1 mm) T°: 35
LC-MS/MS Tran J. [81]	Dried ground inflorescences	CBC(A), CBD(A), CBDV(A), CBG(A), CBL, CBN(A), THCV(A), THCA-A, Δ^8^-THC, Δ^9^-THC	no	8 min Quantification (LOD: 0.1 µg/mL LOQ: 0 08–0.71 µg/mL)	MeOH (73–126%)	Water + formic acid 0.1% ACN + formic acid 0.1% (gradient)	Luna Omega C18 (1.6 µm, 150 mm × 2.1 mm) T°: 40
LC-DAD Wilson W.B. [82]	Oils	CBC, CBD(A), CBDV(A), CBG(A), CBN, THCA, Δ^8^-THC, Δ^9^-THC	no	10 min Quantification (n.m.)	MeOH	ACN Water (isocratic)	ACE 5 C18-AR (5 µm, 250 mm × 0.5 mm)
2021							
HPLC-MS/MS Bueno J. [89]	Dried and ground inflorescences	CBD, Δ^9^-THC, tetrahydrocannabiphorol	Δ^9^-THC-d3	n.m. Quantification (LOD: 0.0008% *w*/*w*)	MeOH:chloroform 9:1 EtOH	Water + 5 mM ammonium formate MeOH (gradient)	Restek Raptor ARC C18 (2.7 µm, 150 mm × 2.1 mm) T°: 45
HPLC-UV (220 nm) Chen X. [72]	Dried and ground inflorescences	CBC, CBD(A), CBDV, CBG(A), CBN, THCA, Δ^9^-THC, Δ^8^-THC, THCV	no	17 min Quantification (LOD: 0.01–0.11 µg/mL LOQ: 0.04–0.36 µg/mL)	MeOH	Water + 0.085% phosphoric acid 0.085 MeOH + 0.085% phosphoric acid (gradient)	NexLeaf CBX Potency C18 (2.7 µm, 150 mm × 4.6 mm) + guard column T°: 50
HPLC-DAD (220 and 240 nm) Madej K. [66]	Oils	CBD(A), CBN, Δ^9^-THC	no	12.5 min Quantification (LOD: 0.17–1.94 µg/mL LOQ: 0.78–5.03 µg/mL)	ACN (69.5–109.5%)	Water + acetic acid 0.5% ACN (gradient)	Spheri-5 C18 (5 µm, 250 mm × 4.6 mm) T°: 25
LC-MS/MS Merone G.M. [90]	Oils	CBD(A), CBN, CBG, THCA, THCV, Δ^9^-THC	CBD-D3, CBN-D3, Δ^9^-THC-D3	15 min Quantification (LOD: 0.01–01 mg/mL LOQ: 0.05–0.1 mg/mL)	Isopropanol	Water + formic acid 0.2% + ammonium formate 2 mL MeOH + formic acid 0.2% + ammonium formate 2 mL (gradient)	Hypersil Gold PFP (1.9 µm, 50 mm × 2.1 mm) n.m.
HPLC-UV (232 nm) Stempfer M. [94]	Oils, dried ground iInflorescences	CBC, CBDA, CBD, CBDV, CBGA, CBG, CBN, Δ^8^-THC, Δ^9^-THCA, Δ^9^-THC	Fencamfamine	30 min Quantification (LOQ (inflorescences): 10–3000 µg/kg)	MeOH:water 1:1	Water + Ammonium formate 20 mM + formic acid 0.1% ACN MeOH + 10 mM ammonium formate + 0.05% formic acid (gradient)	Luna C18 (5 µm, 150 × 4.6 mm) + guard column T°: 40
nanoLC-UV nanoLC-MS Žampachová L. [87]	Ground inflorescences	CBD(A), CBG(A), CBC, Δ^9^-THC, THCA	no	12 min Quantification (LC-UV LOD: 0.125–1.0 µg/mL LOQ: 0.5–2 µg/mL LC-MS: LOD: 0.020–0.125 µg/mL LOQ: 0.055–0.175 µg/mL)	EtOH (80–95%)	ACN + water + formic acid 1% (isocratic)	ChromSpher C18 (3 µm, 150 mm × 0.1 mm) n.m.
2020							
HPLC-UV-MS/MS (235 nm) Nemeškalová A. [97]	Oils, Ground plant material (and other matrices)	CBDA, CBD, CBDV, CBGA, CBG, CBN, THCA, Δ^8^-THC, Δ^9^-THC	CBN-d3, Δ^9^-THC-d3	7.8 min Quantification (oils: LOD: 0.2–1.0 µg/g LOQ: 1–4 µg/g plant material: LOD: 1–5 µg/g LOQ: 5–20 µg/g)	Ethyl acetate:isopropanol 1:1	Ammonium acetate 10 mM in 5% aqueous MeOH + formic acid 0.1% MeOH + ACN (gradient)	Poroshell 120 EC-C18 (2.7 µm, 100 mm × 2.1 mm) + guard column T°: 35
UHPLC-MS/MS Berthold E.C. [98]	Dried, ground flowers (only), composite sample (leaves, flowers, and stems)	CBC, CBL, CBD(A), CBDV, CBG(A), CBN, THCA, Δ^8^-THC, Δ^9^-THC, THCV	Δ^9^-THC-d3, 11-nor-9-carboxy Δ^9^-THC-d9	6 min Quantification (LOD: 1 ng/mL LOQ: ≤ 0.05% *w*/*w*)	MeOH:water 9.5:5	Water + formic acid 0.1% MeOH:CAN (gradient)	UPLC BEH C18 (1.7 µm, 100mm × 2.1 mm) T°: 40
2019							
HPLC-DAD (211 and 220 nm) Burnier C. [52]	Dried ground leaves, flowers	CBN, CBD, THCA, Δ^9^-THC	Tribenzylamine	19.2 min Quantification (LOD: 4.54 µg/mL LOQ: 15.13 µg/mL)	EtOH	Phosphoric acid 50 mM Water + ACN ACN + formic acid 0.1% (isocratic)	Nucleodur C18 gravity (5 µm, 250 mm × 4.6 mm) T°: 35
UHPLC-HRMS/MS Citti C. [85]	Oil	CBG(A), THCA, CBD(A), CBN, CBD, Δ^9^-THC, Δ^8^-THC(And other cannabinoids)	CBD-d3, Δ^9^-THC-d3	65 min Quantification (screening) (n.m.)	EtOH	Water + formic acid 0.1% ACN + formic acid 0.1% (gradient)	Poroshell 120 EC-C18 (2.7 µm, 100 mm × 3 mm) T°: 25
HPLC-UV (220 nm) Mandrioli M. [69]	Dried ground inflorescences	CBD(A), CBG(A), CBC, THCV, Δ^9^-THC, Δ^8^-THC, THCA	no	20 min Quantification (LOD: 0.11–0.17 µg/mL) LOQ: 0.88–3.79 µg/mL-	ACN	Water + 0.085% phosphoric acid ACN + 0.085% phosphoric acid	Nex-Leaf CBX/Potency (2.7 µm, 150 mm × 4.6 mm) + guard column T°: 35
2018							
HPLC-UV Carcieri C. [99]	Oil	CBD(A), CBN, Δ^9^-THC	CBD-d3, Δ^9^-THC-d3	3.5 min Quantification (LOQ: 5 ng/mL)	Isopropanol	ACN:water + formic acid 0.1% Isopropanol:ACN + formic acid 0.1% (gradient)	Acquity UPLC HSS T3 (1.8 µm, 30 mm × 2.1 mm) T°: 30
HPLC-UV Citti C. [86]	Oil	CBD(A), CBN, CBG, CBDV, THCA, Δ^9^-THC	/	15 min Quantification (LOD: 0.2 µg/mL LOQ: 1 µg/mL)	EtOH	Water + formic acid 0.1% ACN + formic acid 0.1% (isocratic)	Poroshell 120 EC-C18 (2.7 µm, 100mm × 3 mm) n.m.

CBC(A): cannabichromen(-ic acid); CBD(A): cannabidiol(-ic acid); CBDV(A): cannabidivarin(-ic acid); CBG(A): cannabigerol(-ic acid); CBL(A): cannabicyclol(-ic acid); CBN(A): cannabinol(-ic acid); CBT: cannabicitran; LOD: limit of detection; LOQ: limit of quantification; Δ^9^-THC(A): Δ^9^-tetrahydrocannabinol(-ic acid); Δ^8^-THC: Δ^8^-tetrahydrocannabinol; THCV(A): tetrahydrocannabidivarin(-ic acid).

### 3.3. Supercritical Fluid Chromatography (SFC)

Since 2010, ultrahigh performance (UHP)-SFC has been used, with some advantages compared to UHPLC. The former allows for a very fast analysis time due to the use of column particles below 2 µm in diameter. Moreover, SFC is an eco-friendly technique that uses supercritical CO_2_ as the mobile phase, which is a gas with a low viscosity and high diffusivity [29]. Generally, these methods require lower amounts of organic solvents since they are mixed with supercritical CO_2_. However, only a few papers describe SFC methods for cannabinoid quantification in cannabis plant materials and CBD oils. Interestingly, in order to demonstrate the advantages of SFC for routine cannabinoid analysis, a comparative study between UHPLC and UHP-SFC techniques hyphenated with a UV detector for cannabinoid quantification in cannabis (plant material) was realized. It was demonstrated that both methods are in accordance [103].

Pilarova et al. developed a UHP-SFC method for the quick determination of 12 cannabinoids in different matrices, including plant materials and oils [104]. This optimized method led to the separation of two groups of isomers (THCA and CBDA for the first group and CBC, CBD, CBL, Δ^9^-THC, and Δ^8^-THC for the second group). Table 5 summarizes two cannabis-related SFC applications [104,105].

### 3.4. Spectroscopy

Spectroscopic techniques are based on the interaction of light with the molecules or the samples under investigation. In the context of the analysis of cannabinoids, especially their analysis in herbal samples, infrared spectroscopy, comprising MIRS, NIRS, and Raman spectroscopy, is the most relevant.

#### 3.4.1. MIRS and NIRS

MIRS and NIRS analysis are non-destructive, fast, and green techniques and have been used across various fields [106]. It was found that NIRS combined with chemometrics had great potential in the analysis of natural plant products. Indeed, moisture, volatile substances, and chemical compounds in herbal products can be analyzed using NIRS [107]. Most papers in this review focused on the analysis of cannabis herbs [105,108,109,110,111,112,113,114,115,116,117,118,119]. Only the papers published by Duchateau C. et al., Chen Z. et al., and Risoluti R. et al. [120,121,122] focused on oils. However, the sensitivity is low for both methods, with values of 0.1% for MIRS and 1% for NIRS [29]. Spectroscopic methods produce highly informative spectra, containing a lot of data that are difficult to interpret [108,123]. Multivariate analysis techniques allow the analysis of large and complex datasets and are better applied to the extraction of the information of interest [108,123]. This combination was already applied to the classification of herbal cannabis samples and the quantification of Δ^9^-THC in cannabis samples for recreational use [123]. Duchateau et al., for example, used both a benchtop FT-NIRS and a hand-held device to discriminate between legal and illegal cannabis samples (dried flowers) based on the European and Swiss legislation, as well as soft independent modeling of class analogies (SIMCA) and partial least squares discriminant analysis (PLS-DA) models [55]. MIRS and calibration techniques were also used to classify different kinds of oil between them [124]. In particular, the use of attenuated total reflectance (ATR) sampling with MIRS is described as one of the main methods used for liquid analysis by infrared spectroscopy [125]. NIR with transmittance, reflectance, and transflectance is a promising option to evaluate, for example, the quality of oilseeds and edible oils [126]. Chen et al. determined CBD in hemp oil by NIRS in reflectance mode coupled to multivariate calibration [121]. Duchateau et al. used both MIRS and NIRS for the identification of the oil matrix for some commercially available CBD oils (on both the regular market and the Internet market) and for the estimation of the level of CBD present. For this, they combined the spectral data with SIMCA and PLS analysis and showed that although MIRS and NIRS gave comparable results for the classification of the oil matrices, MIRS was the most valuable for the estimation of the CBD content [120].

It has been noticed that considerable attention has been given to the miniaturization of spectroscopic devices for on-site measurements. Hand-held devices offer interesting possibilities by allowing on-site analysis by reducing the size of the device. These properties allow cost reductions and easy transport. Moreover, they offer accurate measurements and reliable high performance [127]. As an example, Risoluti et al. have developed a screening test for the real-time detection of cannabinoids in hemp flour using a miniaturized analytical platform based on a MicroNIR spectrometer [108]. In addition, Zimmerleiter et al. developed a compact sensor based on NIR spectroscopy to differentiate between legal and illegal cannabis samples according to their Δ^9^-THC content. The spectral data here were treated using PLS-DA [119]. Tran et al. can distinguish between high-THCA and even-ratio chemovars in a glasshouse environment thanks to their method [109].

Table 6 provides an overview of cannabis-related NIRS and MIRS applications.

#### 3.4.2. Raman Spectroscopy

As for MIRS and NIRS, Raman spectrophotometers can be used in the lab with benchtop devices or on-site with hand-held devices, and current in situ analysis can be performed without sample pretreatment [128]. However, due to the high fluorescence produced by chlorophyll b and carotenoids, Raman spectroscopy is less frequently investigated for the analysis of herbal materials than for the characterization of drugs of abuse [128]. Actually, the Raman detector is unable to distinguish between the light emitted from Raman scattering and the fluorescence. This means that fluorescence may interfere with the Raman spectrum, since fluorescence peaks are wider and greater than Raman peaks. To avoid fluorescence, the wavelength of the laser must be adapted, by employing, for instance, an infrared laser (1064 nm) [129]. Porcu et al. developed a rapid in situ detection method for CBD and Δ^9^-THC in cannabis (plant material) with a Raman spectroscope containing such an infrared laser (1064 nm). It was demonstrated to be able to discriminate dried inflorescences of CBD-rich and Δ^9^-THC-rich cannabis based on the Δ^9^-THC and CBD contents, but it required the selection of the glandular trichomes of the sample with a microscope [130]. An overview of cannabis-related Raman applications in the field is given in Table 7.

The strength of the Raman effect itself is another restriction because only 0.0000001% of the scattered light is Raman scattering. Indeed, most light that is scattered off a substance will be Rayleigh scattered light. Raman spectroscopy relies on detecting Raman scattered light and its sensitivity is therefore limited. The Raman approach can detect as low as 1 ppm of Δ^9^-THC and 65 ppb of CBN. Similar results were found for seven other cannabinoids analyzed by Grijalva et al. [131]. They have shown the robustness and reliability of results obtained by Raman spectroscopy hyphenated with chemometrics and machine learning [131]. They have used the density functional theory (DFT) to attribute spectral features in cannabinoids, such as in Wolfe et al. [132].

The sensitivity of the technique can be substantially improved using surface-enhanced Raman spectroscopy (SERS). In contrast to conventional Raman spectroscopy, it consists of the absorption of the analyte on roughened metallic surfaces (e.g., gold and silver colloids) to increase the Raman scattering by up to 1000 times [129,133]. However, compared to classical Raman spectroscopy, SERS is a destructive technique.

An analysis of Δ^9^-THC and its analogs using SERS was developed by Islam et al. [134]. Two years later, Botta et al. developed an approach for the trace analysis of Δ^9^-THC and CBN detection [135]. They describe the fabrication of several SERS substrates (Ag- nanorods) to optimize the method and observed that the fabrication of the nanorods must be reproducible for reliable results to be obtained.

Another spectroscopic approach was presented by Gilmore et al. [136]. They developed an approach based on absorbance, transmittance, and fluorescence excitation-emission matrix (A-TEEM) spectroscopy to differentiate between different chemotypes and to quantify the main cannabinoids, Δ^9^-THC and CBD. Therefore, spectroscopic measurements were performed on extracts in MeOH/dichloromethane (9:1), followed by the development of classification models, using principal component analysis (PCA) and extreme gradient boosting (XGB) discriminant analysis for chemotype classification and XGB regression for the quantification of Δ^9^-THC and CBD. Models were built using GC-FID and HPLC-UV results as reference data.

**Table 7:** Raman spectroscopy applications: overview of the literature.

Spectroscopic Technique (Reference Method) 1st Author [Reference]	Matrix	Cannabinoids	Instrumentation Spectrometer Type	Laser source Resolution Acquisition Time Laser Power	Chemometric Model or Spectral Analysis (Spectral Range) (Preprocessing)
2024					
Raman Grijalva J. [131]	Standards	CBD(A), CBC, CBG, CBN, Δ^9^-THC, THCA,	Raman microscope	785 nm n.m. 10 s 100 mW	Linear DA DFT (400–2200 cm^−1^)
2023					
Raman Wolfe T.J. [132]	Dried ground buds (different cultivars of cannabis) to isolate phytocannabinoids	CBC, CBD, CBG(A), CBN, Δ^9^-THC	Raman equipped with camera	532 nm n.m. 2 s	DFT (200–4000 cm^−1^) /
2022					
Raman (GC-FID) Porcu S. [130]	Not dried, not ground inflorescences	CBD, Δ^9^-THC	Raman spectrometer and stereomicroscope equipped with camera	1064 nm /	PCA Discrimination (655–1800 cm^−1^) /
Raman-SERS Botta R. [135]	Standards	CBN, Δ^9^-THC	Raman microscope	785 nm / 10 s 20 mW	/ (620–1720 cm^−1^) /
2020					
Raman Sánchez L. [137]	Dried flowers	/	Hand-held	831 nm 15 cm^−1^ 10 s 495 mW	SIMCA Orthogonal PLS-DA Discrimination (701–1700 cm^−1^) SNV, 1st derivative
Raman-SERS Islam S. [134]	Standard	CBD, CBN, Δ^9^-THC	Raman microscope	633 nm 0.02–05 cm^−1^ 10 s 10 mW	/

CBC: cannabichromen; CBD(A): cannabidiol(-ic acid); CBG(A): cannabigerol(-ic acid); CBN: cannabinol; DA: discriminant analysis; SNV: standard normal variate; Δ^9^-THC(A): Δ^9^-tetrahydrocannabinol(-ic acid).

## 4. Discussion

From the studied literature, slightly less than 40 papers using LC, slightly more than 25 papers using GC, and 25 papers using spectroscopic techniques, including NIRS, MIRS, and Raman spectroscopy, were retained. It is clear that chromatographic techniques, such as GC and LC, are the most popular in the context of cannabinoid analysis in cannabis herbs and oily products. However, most of the papers (>70%) are about cannabis herbs, with less than half on oils.

GC (hyphenated with FID) was somewhat considered the gold standard as it is used in the EU’s official method for the analysis of agricultural hemp. However, other official methods such as AOAC [26] use other techniques such as liquid chromatography (generally hyphenated with DAD) for detecting and measuring cannabinoids in hemp. The reason to prefer LC vs. GC can be notably explained by the limitations of GC.

The choice of LC or GC for this type of analysis is a subject of discussion in the literature, especially in the context of herbal smoking products, where the content of Δ^9^-THC should be checked, in order to verify its compliance with the legislation, as well as the content of CBD in order to check label compliance. Duchateau et al. [78] compared the performance of the GC-FID method and an LC-UV method in performing an analysis of CBD and Δ^9^-THC in herbal products and concluded that very similar results could be obtained. Although not statistically significant, it was observed that LC tends to overestimate the content while GC had a tendency to underestimate it. This is generally not a problem, except when the Δ^9^-THC content flirts with the legal limits. The underestimation in GC could be explained by thermal degradation during sample injection in the GC injector port. Garcia-Valverde et al. have demonstrated that CBD can be degraded into Δ^9^-THC, which is subsequently converted into CBN, and CBC can emerge as a degradation product of CBG [138]. Here, analysis certificates based on LC could give different results to the ones obtained by controlling agencies performing the analysis with GC. It should be kept in mind that in GC, the decarboxylation of the acidic forms should be complete and this can be influenced by several factors, so this step should be very well-validated [139]. On the other hand, in LC, acidic and decarboxylated forms are quantified separately, with measurement uncertainties playing for both compounds, which results in higher uncertainties when total THC is calculated to check compliance with the legislation. In the opinion of the authors, the only possible way to solve the problem of discrepancies is to impose a “standard method”. In this way, producers, distributors, and authorities will all evaluate the product in the same way, avoiding unnecessary legal procedures and the loss of resources. This was, for example, done by the European Pharmacopoeia, which developed and validated an LC method for the quantification of total Δ^9^-THC and total CBD for cannabis flowers, which are used for medicinal purposes [140].

The use of mass spectrometry is gaining importance, especially due to its ability to quantify very low amounts of cannabinoids. In addition, the distinction between structurally very closely related molecules is a huge advantage. Moreover, methodologies capable of providing information on both terpenes and cannabinoids in different matrices are deemed necessary. The extension of the number of cannabinoid molecules to be analyzed, as well as the fact that the acidic forms can be quantified separately, explains the rising popularity of LC-MS/MS.

It also has to be emphasized that, as for all analytical methods, robust validation is necessary and should cover the whole process, as well as the range of matrices to be covered. Some articles were published with methods developed and validated using only reference standards of a series of cannabinoids [141,142]. These papers have their value, e.g., in optimizing the separation of the strongly structurally related cannabinoids [141] or even in the use of experimental design for method development or optimization [142], though for practical applications in the context of quality control and market surveillance, more thorough validation, including of matrix effects and interferences, is pivotal.

This review was limited to the analysis of herbal products and the so-called CBD oils, which are used in a (para-)pharmaceutical context or as herbal smoking products. However, cannabis, cannabinoids, and cannabis extracts can be found in a wide range of products nowadays, as already summarized in Table 1. Contrary to herbal matrices, a lack of regulation exists, with no mandatory analytical controls for cannabis-based products, leading to uncertainty about the composition and quality of the products offered to consumers [143]. For such a high diversity of products, the applicability of analytical methods, as well as the validation strategies applied, may differ and therefore all analytical scientists involved in the development of methods for cannabinoids and in market surveillance should be aware that each matrix has its own characteristics and its own analytical challenges, resulting in methods that are not always transferable from one domain to another. Analytical approaches for cannabinoid analysis in cosmetics, vaping products, and food are a high-interest topic, as illustrated by recent reviews [144,145] and by the publication of newly developed methods [146,147,148]. Recently, the Food and Drug Administration (FDA) pointed out that CBD has raised various safety concerns with long-term use. Customers would benefit from a new regulatory approach to manage and reduce the dangers associated with CBD products [149]. This should hopefully lead to the development of new official methods for analyzing CBD-containing products.

Spectroscopic techniques, especially MIR and NIR, were often applied for this type of analysis and showed promising results when multivariate analysis techniques were used for data treatment and interpretation. Spectroscopic techniques, however, are less sensitive and suffer from the fact that no separation of the different molecules occurs. On the other hand, spectroscopy has a huge advantage considering its speed of analysis, portability, and environmentally friendly nature.

Two other methods covered in this review are SFC and Raman spectroscopy. The use of SFC for cannabinoid analysis is still being developed. For the moment, this technique is less encountered in laboratories, dealing with cannabinoids, and also shows a similar selectivity and sensitivity as classical LC. SFC may become more important in the future, especially during the transition to more environmentally friendly methods and techniques and green chemistry in general. Raman spectroscopy is influenced by fluorescence and is less sensitive. Raman combined with SERS can be a solution to increase its sensitivity. However, the device is not yet well-exploited and the creation of nanoparticles, as well as the reproducibility of these, is not yet optimal.

Table 8 summarizes the different advantages and disadvantages of the chromatographic and spectroscopic techniques discussed in this review. Chromatographic techniques can be more easily automatized than spectroscopic techniques, yet, the time required for analysis is higher. Liquid chromatography-based methods can distinguish between acidic and decarboxylated forms. However, the temperature and the flow rate influence the decarboxylation/degradation of cannabinoids and the separation of the analytes. Due to the ease of use and the low analysis cost of spectroscopic techniques, these are less sensitive and generally need multivariate modeling to distinguish samples and predict the concentrations of analytes.

Next to the methods reviewed in this paper, some alternative methods were encountered, for which only one or a few papers were published. For the moment, these techniques are not ready yet for implementation as a routine analysis technique since more research will be necessary. One of these techniques is electrochemistry. Although cannabinoids are electrochemically active, only a few papers can be found in the literature for the analysis of cannabis (herbal plants and oils). This could be explained by the oxidation potentials of Δ^9^-THC and CBD, which are similar, requiring the combination of electrochemistry with another (preferably separation) technique to solve this problem. Despite the challenges, Deenin et al. developed an electrochemical lateral flow device to detect THC in 2023 [150]. The concept of the method is that Δ^9^-THC in the sample is immunocomplexed with a ferrocene carboxylic acid-labeled antibody, which binds to the immobilized cannabinoid receptor 2 above the electrode. They have applied their method on dried cannabis samples to prove its ability to quantify Δ^9^-THC and total THC [150]. Huang et al. presented another alternative, namely the use of cyclic ion mobility combined with a QToF mass spectrometer. Ion mobility could be an alternative to chromatography. Huang et al. were able to differentiate and quantify a series of cannabinoids, including acidic forms and structural isomers of Δ^9^-THC. These alternatives suggest again that cannabis and cannabis-derived products can be analyzed using a wide variety of techniques and approaches, and that in the context of quality control, label accuracy checks, and market surveillance, there is an urgent need for standardization [151].

This review was limited to the analysis of cannabinoids, with a primary focus on quality control and market surveillance. The focus on cannabinoids is inspired by the fact that these are considered the active compounds to which pharmacological effects are designated. Recent research revealed that there could be a synergetic effect between cannabinoids and other phytochemicals, for example, terpenes. If this is confirmed, a series of new analytical methods able to analyze both terpenes and cannabinoids simultaneously will be published. In fact, at the moment, the rising interest in terpenes has already resulted in different papers [40,65,88].

## 5. Conclusions

The expansion of analytical techniques for cannabinoid detection or quantification has been due to the advent of cannabis-based (consumer) products on the market, as well as the substantial advancements in medicinal and agronomic research. In the period reviewed (2018–November 2024), GC and LC have been widely applied, often using similar methods. Low-Δ^9^-THC products are not controlled under drug laws. Authorities aim to develop techniques to check the legality of these products in order to prevent the legislation from being circumvented. For the moment, Δ^9^-THC and CBD are the main molecules of interest for regulatory bodies. However, due to continuous research and commercial value, more and more naturally occurring cannabinoids such as CBG, CBC, CBDV, and THCV are becoming of interest and are, whether justifiably or not, linked to several health claims. This means that in the future, more and/or new cannabinoids will have to be analyzed in different matrices [97]. Examples of such new cannabinoids are epicannabidiol hydrate [152] or tetrahydrocannabihexol acetate [153]. The design of experiments answers the request of a growing scientific interest in *Cannabis sativa* L., which aims to develop or optimize with minimum experimental trials [154].

It can be concluded that the market for cannabis-based products is still growing and diversifying, requiring constant updates to existing methods and the development of methods adapted to new matrices. The growing and diversifying market also requires some standardization in the form of legislation or guidelines in order to prevent inconsistencies between the results provided by the producers and distributors and the ones obtained by the controlling agencies, as has been seen over the past years for herbal smoking products [78]. In order to control the market and protect consumers, there is a need for official methods for the products at risk, as exists for agricultural hemp and for cannabis flowers used as an active pharmaceutical ingredient [140]. Alternatively, guidelines through official bodies could be established together with the industry to agree on the criteria these products should respond to and how to check them. This also needs to include the validation protocol. Indeed, in the papers cited in the current review, different method validation guidelines are used, e.g., SWGTOX guidelines [95,155], US FDA, bioanalytical method validation guidelines for industry (ICH) [156], and ISO 17025 [78]. It is important to compare the same parameters of validation of two methods based on the same criteria. When deciding and creating these norms and the standardized analytical protocol, the possibility of using green techniques, such as spectroscopy, or more environmentally friendly techniques, should be explored. Of course, the primary goal is to protect the consumer, but the principles of green chemistry are finding their way and will add to the protection of consumers and, more broadly, the population, especially when they are embraced by the authorities and other official regulatory bodies.

## Figures and Tables

**Table 1:** The chemical structures of some main phytocannabinoids (and (in red) the carboxylic precursor).

Cannabinoid	Structure
Cannabichromen(-ic acid) CBC(A)	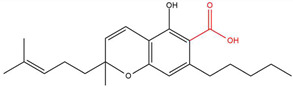
Cannabidiol(-ic acid) CBD(A)	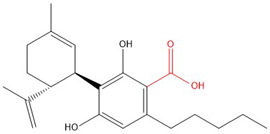
Cannabidivarin(-ic acid) CBDV(A)	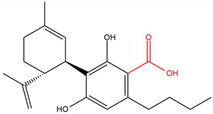
Cannabigerol(-ic acid) CBG(A)	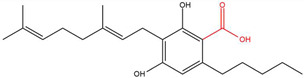
Cannabinol(-ic acid) CBN(A)	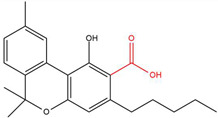
Delta-9-tetrahydrocannabinol(-ic acid) Δ^9^-THC(A)	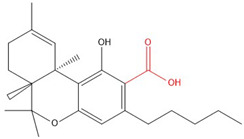
Tetrahydrocannabidivarin(-ic acid) THCV(A)	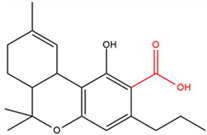

**Table 2:** Overview of low-Δ^9^-THC products circulating on the European market.

Product	Description and Information About the Product
Herbal product and resin for smoking	*Cannabis* spp. flowers (CBD cultivars)
e-liquids	Liquid containing CBD put in an e-cigarette (vaping product)
Crystals	Solid containing CBD used to make the e-liquid itself
CBD oil (internal use)	Oil (e.g., hemp seed oil) + CBD
Edible products—Food	Food based on *Cannabis sativa* L. (CBD cultivars) (e.g., cookies, chocolate, and pasta)
Food supplements	e.g., Capsules, gummies, and beverages containing CBD
Hemp seed oil	Oil made from whole seeds
Herbal tea	*Cannabis* leaves/flowers destined to be infused
Cosmetics	e.g., balms, shampoos, oils, and creams containing CBD
Potpourri	*Cannabis* spp. flowers (CBD cultivars)

**Table 5:** SFC applications: overview of the literature.

Analytical Technique Year 1st Author [Reference]	Matrix (Sample State)	Cannabinoids	Analysis Time (LOD/LOQ)	Solvent Extraction	Mobile Phase	Column
UHP-SFC-MS/MS Pilařová V. 2022 [104]	Oil, dried plant material (herbs, leaves, and flowers), and other matrices	CBC, CBDA, CBD, CBDV, CBGA, CBG, CBL, CBN, THCA, Δ^8^-THC, Δ^9^-THC, THCV	3.5 min (n.m.)	ACN (plant material) EtOH (dietary supplements, cosmetics)	CO_2_ + MeOH: ACN (2.5:7.5) + 5% water	Waters Acquity Viridis 2-Ethylpyridine, C18 (1.7 µm, 100 × 3.0 mm)
UHP-SFC-UV Deidda R. 2020 [105]	Ground inflorescences, resins	CBC, CBDA, CBD, CBGA, CBG, CBN, THCA, Δ^8^-THC, Δ^9^-THC	6 min (LOD: 1.5–2.30 µg/mL LOQ: 2.96–4.55 µg/mL)	EtOH	CO_2_ + MeOH:ACN (2.5:7.5) (Gradient)	Waters Acquity UPC Torus DIOL (1.7 µm, 100 × 0.3 mm)

CBC(A): cannabichromen(-ic acid); CBD(A): cannabidiol(-ic acid); CBDV(A): cannabidivarin(-ic acid); CBG(A): cannabigerol(-ic acid); CBL: cannabicyclol; CBN(A): cannabinol(-ic acid); LOD: limit of detection; LOQ: limit of quantification; Δ^9^-THC(A): Δ^9^-tetrahydrocannabinol(-ic acid); Δ^8^-THC: Δ^8^-tetrahydrocannabinol; THCV: tetrahydrocannabidivarin.

**Table 6:** NIRS and MIRS applications: overview of the literature.

Spectroscopic Technique (Reference Method) 1st Author [Reference]	Matrix (Sample State)	Cannabinoids	Instrumentation Spectrometer Type	Sample Handling Resolution Scans	Chemometric Model (Model Evaluation Metrics) (spectral Range) Preprocessing
2024					
NIR (GC-FID, LC-DAD) Zimmerleiter R. [119]	Dried, ground (with different degrees of fineness) inflorescences	THC total	Hand-held	Diffuse reflectance n.m. 20	PLS-DA Discrimination (ccr : 70.4–78.6%) (1550 nm-1950 nm) Smoothing and 1^st^ derivative (Savitzky-Golay) + SNV
MIR and NIR (GC-MS) Duchateau C. [120]	Oils	CBD	Benchtop FT	ATR-MIR 4 cm^−1^ 32 Transflectance (NIR) 8 cm^−1^ 16	SIMCA (ccr: 100%) PLS-R (RMSEC: 1.0–4.4 RMSEP:0.9–3.9) (5000–16000 nm) (1600–2500 nm) Smoothing and 2^nd^ derivative (Savitzky-Golay)
NIR (LC-MS) Tran J. [109]	Dried ground inflorescences	THCA	Hand-held (MicroNIR)	Diffuse reflectance	PLS-DA (RMSEC: 0.15 RMSEP: 0.12) PLS-R (RMSEC: 26.34–28) RMSEP: 21.49–23.49) SVM-R (RMSEC: 23.87–25.11 RMSEP: 22.49–24.87) XGB-R (RMSEC: 0.02–12.27 RMSEP: 23.02–28.77) (10,526–6060 cm^−1^) 2nd derivative, SNV, MC
2023					
NIR (HPLC-UV) Gloerfelt-Trap F. [110]	Dried ground aerial part	CBC, CBDA, CBD, CBDVA, CBDV, CBGA, CBG, CBN, THCA, Δ^9^-THC, THCVA, THCV	Hand-held n.m.	Diffuse reflectance /	Cross-validation (RMSE: 5.27–247.66) Hold-out validation (RMSE: 18.54–94.5) (1350–2500nm) 1st derivative, order 1
NIR Tran J. [111]	Dried ground inflorescences	CBCA, CBC, CBDA, CBD, CBDVA, CBDV, CBGA, CBG, CBNA, CBN, THCA, Δ^9^-THC, THCVA, THCV	Benchtop FT Hand-held (Micro) n.m.	Diffuse reflectance 16 cm^−1^ 64 Diffuse reflectance / 100	PCA PLS-DA (FT: RMSEC: 0.123–0.237 RMSEP: 0.106–0.211 Micro: RMSEC: 0.165–0.391 RMSEP: 0.125–0.368) PLS-R (FT: RMSEC:0.07–6.93 RMSEP: 0.06–5.51) (1111–2500 nm) SNV, normalization, detrend, 1st/2nd derivatives
2022					
NIR (HPLC-DAD) Birenboim M. [112]	Dried ground inflorescences	CBCA, CBC, CBDA, CBD, CBGA, CBG, CBL, THCA, Δ^9^-THC, THCV	Benchtop FT	Reflectance 4 cm^−1^ 16	PLS-DA (RMSEC: 0.136–0.232 RMSEP: 0.127–0.228) PLS-R (RMSEC: 0.0086–0.944 RMSEP: 0.011–1.275) (1000–2500 nm) SNV, MSC, normalization (mean centering, autoscaling) GLS, smoothing,
NIR (GC-FID) Su K. [113]	Dried ground plant material	CBD, CBG, CBN, Δ^9^-THC	Benchtop n.m	/ Reflectance or transflectance /	PLS-R (RMSEC :0.01–1.16 RMSEP: 0.01–1.28) (950–1650 nm) /
NIR (HPLC-MS/MS) Yao S. [114]	Dried ground plant material	CBDA, CBD, THCA, Δ^9^-THC	Hand-held (Micro) FT	Diffuse reflectance /	PLS-R (RMSECV: 0.02–0.54 RMSEP: 0.02–0.061) (1350–2560 nm) 2nd derivative (Savitsky-Golay), MC
NIR (HPLC-UV) Jarén C. [115]	Dried ground plant material	CBD, Δ^9^-THC	Hand-held Dispersive	Reflectance / 50	PLS-R (RMSEC: 0.010–0.011 RPD: 2.04) (1200–2200 nm) Normalization, SNV, MSC, SNV-DT, 1st and 2nd derivative (Savitzky-Golay)
2021					
MIR (LC-MS/MS) Cirrincione M. [116]	No dried and no ground inflorescences	CBD(A), CBG(A), CBN, THCA, Δ^9^-THC	Benchtop FT	ATR 4 cm^−1^ 20 scans	PLS-R (RMSEC: 0.163 x10–8–0.238) (4000–400 cm^−1^) 1st derivative: Δ^9^-THC: 1514–1485 cm^−1^ THCA: 141–1391 cm^−1^ CBD: 3085–3060 cm^−1^ CBDA: 982–959 cm^−1^ CBG: 844–830 cm^−1^ CBGA: 820–807 cm^−1^ CBN: 910–872 cm^−1^
NIR Chen Z. [121]	Oils	CBD	Benchtop FT	Reflectance 4 cm^−1^ 64	PLS-R (RMSEC: 5.6 RMSEV: 6.87) SOSVEN (RMSEC: 5.1 RMSEP: 6.6) (1111–2222 nm) 1st derivative (Savitzky-Golay)
NIR (HPLC-UV) Deidda R. [105]	Inflorescence and resin through a plastic bag	THCA, Δ^9^-THC	Hand-held (1) Dispersive Hand-held (2) (Micro) Dispersive	Reflectance Reflectance	PLS-R (Instrument (1) RMSEC: 0.88–1.74 RMSEP: 1.55–2.07) (Instrument (2) RMSEC: 0.74–1.02 RMSEP: 1.04–1.75) (900–1700 nm) (1) (950–1650 nm) (2) 2nd derivtive (Savitzky-Golay), SNV
NIR (HPLC-UV) Geskovski N. [117]	Dried ground flowers and extracts	CBDA, CBD, THCA, Δ^9^-THC	Benchtop FT	ATR 4 cm^−1^ n.m.	PLS-R (extracts (RMSECV: 2.62–5.25) RMSEP: 1.44–3.79 Flowers: RMSECV: 1.41–1.53 RMSEP:1.33–2.32) (5555–25000 nm) Smoothing and 2nd derivative (Savitzky-Golay)
2020					
NIR (GC-MS) Risoluti R. [122]	Oil	CBD, THCA, Δ^9^-THC	Hand-held (Micro) Dispersive	Reflectance 6.25 nm	PLS-DA (RMSEC: 0.001–0.002 RMSECV: 0.003–0.005) (900–1700 nm) Baseline corrected, SNV
NIR (GC-MS) Risoluti R. [108]	Dried inflorescences	CBD, THC total	Hand-held (Micro) Dispersive	Reflectance	PLS-DA PLS-R (RMSEC: 0.003–0.005 RMSEP: 0.005–0.007) (950–1650 nm) 2nd derivative, SNV Different regions of interest
NIR (GC-FID) Duchateau C. [55]	Dried and crushed (by hand) inflorescences	CBD, THC total	Benchtop FT (1) Hand-held (Micro) Dispersive	Reflectance 8 cm^−1^ 16 Diffuse reflectance 11 cm^−1^ 5	SIMCA (Instrument (1) CV ccr: 89–92 External validation ccr: 80–1 Instrument (2) CV ccr: 95–97 External validation ccr :84–93) PLS-DA (Instrument (1) CV ccr: 92–97 External validation ccr: 84–91 Instrument (2) CV ccr: 98–99 External validation ccr :88–95) k-NN (1600–2500 nm) 1st derivative, 2nd derivative, SNV
2018					
NIR (GC-FID) Sanchez-Carnero Callado C. [118]	Dried leaves and flowers ground into a powder	CBC, CBD CBDV, CBG, CBN, Δ^8^-THC, Δ^9^-THC, THCV	Hand-held (1) Dispersive Benchtop (2) FT	Reflectance n.m. n.m. Diffuse reflectance 8 cm^−1^ 32	PLS-R (Instrument (1) RMSEC: 0.02–0.58 RMSEP: 0.03–1.72 Instrument (2) RMSEC: 0.02–0.49 RMESP: 0.04–1.79) (400–2498 nm) (1) (800–2500 nm) (2) Several regions of interest Normalization, 1^st^ derivative, MSC

ATR: attenuated total reflectance; CBC(A): cannabichromen(-ic acid); CBD(A): cannabidiol(-ic acid); CBDV(A): cannabidivarin(-ic acid); CBG(A): cannabigerol(-ic acid); CBN: cannabinol; ccr: correct classification rate (%); FT: Fourier-transform; GLS: generalized least squares; MC: mean centering; MSC: multiplicative scatter correction; n.m.: not mentioned; PCA: principal component analysis; PLS(-R): partial least square (regression); RMSEC: root mean square error of calibration; RMSECV: root mean square error of cross-validation; RMSEP: root mean square error of prediction; RPD: ratio of prediction to deviation; SNV: standard normal variate; SNV-DT: standard normal variate with detrending; SOSVEN: Self-Optimizing Support Vector Elastic Net; SVM-R: Support vector machine in R; Δ^9^-THC(A): Δ^9^-tetrahydrocannabinol(-ic acid); Δ^8^-THC: Δ^8^-tetrahydrocannabinol; THCV(A): tetrahydrocannabidivarin(-ic acid); XGB-R: extreme gradient boosting.

**Table 8:** Comparative table of the specificities of chromatographic and spectroscopic techniques. (+) and (-) signs determine the strengths and weaknesses of each parameter. The number of signs determine the intensity of strengths and weaknesses.

	Chromatographic Techniques			Spectroscopic Techniques		
	GC	UHPLC	UHP-SFC	NIR	MIR	Raman
Automatization	+++	+++	+++	---	---	---
Speed of analysis	--	-	-	+++	+++	++
Parameters of influence	temperature and flow rate	temperature and flow rate	temperature and flow rate	temperature	temperature	temperature
Compounds/samples	decarboxylation of acidic form	+++	+++	no difference between acidic and neutral forms	no difference between acidic and neutral forms	no difference between acidic and neutral forms
Separations	+++	+++	+++	---	---	---
Analyte detection	+++	+++	+++	classification/ prediction	classification/ prediction	classification/ prediction
Sensitivity	+++	+++	+++	--	-	--
Analysis cost	---	---	---	+++	+++	+++
Intuitiveness	-	-	---	+	+	+/-
Handling	---	---	---	+++	++	++
Sample preparation	---	---	---	+++	+++	++
Green analytical chemistry	+/-	+/-	+/-	+++	+++	+++

## Data Availability

Not applicable.
